# Inclusion of progression criteria in external randomised pilot trials: a cross-sectional study of funding applications submitted to the NIHR Research for Patient Benefit Programme

**DOI:** 10.1186/s13063-022-06868-8

**Published:** 2022-11-08

**Authors:** Katie Mellor, James Harwood, Jennie Hejdenberg, Ben Morgan, Susan J. Dutton, Sally Hopewell

**Affiliations:** 1 Oxford Clinical Trials Research Unit/Centre for Statistics in Medicine, Nuffield Department of Orthopaedics, Rheumatology and Musculoskeletal Sciences, University of Oxford, Oxford, OX3 7LD UK; 2 EQUATOR Centre UK, Centre for Statistics in Medicine, Nuffield Department of Orthopaedics, Rheumatology and Musculoskeletal Sciences, University of Oxford, Oxford, OX3 7LD UK; 3 National Institute for Health Research Central Commissioning Facility, Twickenham, TW1 3NL UK

**Keywords:** Pilot trials, Randomised controlled trials, Feasibility studies, Progression criteria

## Abstract

**Background:**

External randomised pilot trials aim to assess whether a future definitive randomised controlled trial (RCT) is feasible. Pre-specified progression criteria help guide the interpretation of pilot trial findings to decide whether, and how, a definitive trial should be conducted. We aimed to examine how researchers report and plan to assess progression criteria in external pilot trial funding applications submitted to the NIHR Research for Patient Benefit Programme.

**Methods:**

We conducted a cross-sectional study of progression criteria inclusion in Stage 1 (outline) and corresponding Stage 2 (full) funding applications for external randomised external pilot trials submitted to NIHR RfPB between July 2017 and July 2019.

**Results:**

Of the 100 Stage 1 outline applications assessed, 95 were eligible for inclusion (of these, 52 were invited to Stage 2 full application; 43 were rejected) and 49/52 were eligible for inclusion at Stage 2 full application (of these, 35 were awarded funding; 14 were rejected). Over half of applications assessed at Stage 1 (48/95, 51%), and 73% of those assessed at Stage 2 (36/49) included progression criteria in their research plans. Progression criteria were most often reported in a stop-go format, often with additional specified factors that should be considered when determining feasibility (Stage 1 33/48, 69%; Stage 2 21/36, 58%). Recruitment and retention were the most frequent indicators of feasibility to inform progression criteria. One-third of applications provided some justification or rationale for their targets (Stage 1 16/48, 33%; Stage 2 12/36, 33%). Funding committee feedback mentioned progression criteria in over 20% of applications (Stage 1 22/95, 23%; Stage 2 11/49, 22%) to either request the addition of progression criteria or provide justification for the criteria stipulated.

**Conclusions:**

Our findings indicate that researchers do not always include progression criteria in external randomised pilot trial applications submitted to research funders. This can result in a lack of transparency in the assessment of randomised pilot trial feasibility.

**Trial registration:**

Open Science Framework osf.io/89ap7, registered 29th June 2021.

## Background

External randomised pilot trials (or pilot RCTs) are small standalone studies that aim to assess the feasibility of a future definitive randomised controlled trial (RCT). They question whether the definitive trial *can be done*, *should we proceed with it*, *and if so*, *how* [1]. Randomised pilot trials are a type of feasibility study that can be identified by their ‘piloting’ feature, where all or part of the future trial is piloted on a smaller scale [1]. This allows researchers to assess any uncertainties that they have about their definitive trial design in advance, and make changes as required. Pilot trials also provide the opportunity for researchers to abandon or not pursue their definitive RCT where significant uncertainties about feasibility remain. This saves time and resources that would otherwise be spent on RCTs that are unlikely to be successful.

How researchers plan to interpret their pilot trial findings to determine trial feasibility should be built into the study design from the earliest opportunity, i.e. during protocol development and pilot trial funding application. Progression criteria are pre-specified targets, based on the feasibility objectives of the pilot trial, that help guide the interpretation of pilot trial findings to decide whether, and how, a definitive trial should be conducted.

One of the largest funders of pilot and feasibility studies in the UK is the National Institute for Health Research (NIHR) Research for Patient Benefit (RfPB) funding stream. The NIHR RfPB programme published guidance on applying for feasibility studies in 2017 (v1.0, July 2017) to stipulate that applications for feasibility trials should include clear progression criteria. This was updated in 2021 (v2.0, February 2021) to cover all types of preparatory studies and included the expectation that the application would include progression criteria and set out the pathway to RCT [2]. Pre-specifying clear progression criteria facilitate the transparent assessment of pilot trial feasibility and limit the potential for research waste where unfeasible pilot trials progress to unfeasible RCTs [3], and feasible pilot trials do not progress to further research [4].

Previous studies have investigated the use of progression criteria in NIHR Health Technology Assessment (HTA) funded RCTs with internal pilot phases [5, 6]. These studies found that progression criteria are often specified for trials with an internal pilot, but individual criteria targets vary, and rationale is often not given. They also highlighted increasing preference towards the traffic light or stop-amend-go format that is also recommended for progression criteria for internal pilot trials [7]. Although previous research has highlighted insufficient reporting of progression criteria in external pilot trial protocols [8], a review of stipulated progression criteria in external pilot trial funding applications has not previously been conducted.

The aims of this study were to examine the progression criteria stipulated in the research plans of NIHR RfPB funding applications to identify how researchers conducting randomised pilot trials plan to determine the feasibility of a future definitive RCT. Our primary objective was to examine how researchers report and plan to assess progression criteria in external pilot trial funding applications submitted to NIHR RfPB. Our secondary objectives were to determine which indicators of feasibility inform progression criteria, to document and describe any rationale provided for stated progression criteria, and to determine the extent and context in which progression criteria are mentioned in RfPB committee feedback provided to the researchers.

## Methods

A protocol for this study is registered on the Open Science Framework (osf.io/89ap7) [9]. The University of Oxford Medical Sciences Interdivisional Research Ethics Committee have approved this research (R74410/RE001). A Data Sharing Agreement between the Secretary of State for Health and Social Care and the Chancellor, Masters and Scholars of the University of Oxford was agreed and signed prior to data sharing.

### Sample of included applications

The research plans of Stage 1 outline NIHR RfPB funding applications for external randomised pilot trials, with a funding decision between July 2017 and July 2019, were eligible for inclusion, irrespective of whether they were subsequently funded. This date range was chosen because guidance for the inclusion of progression criteria in RfPB applications was first published in July 2017. Where Stage 1 application outlines had been invited to full Stage 2 application, the corresponding full Stage 2 application research plan was included. Committee feedback at both Stage 1 outline and full Stage 2 application (if applicable) was also included.

### Database search and retrieval of applications

The RfPB database was searched by a NIHR RfPB Senior Programme Manager to identify all Stage 1 application outlines with a funding decision made between July 2017 and July 2019. Application titles and plain English summaries were searched for the keywords: ‘pilot’, ‘feasibility’ or ‘feasible’. A second keyword search for ‘random*’ in the title or plain English summary was used to identify those with a randomised design. To ensure all randomised pilot trials had been identified, the research plan section of the applications was also searched for the terms ‘random’ or ‘control’.

NIHR RfPB Programme Managers obtained approval from researchers for their application to be included and shared with the research team at the University of Oxford. Once approval was obtained any identifiable information was redacted before the application research plans were shared via secure encrypted transfer. Each redacted application was given a unique ID which was maintained throughout data collection. Each application was reviewed upon receipt to provide a second confirmation that the research plan described an external randomised pilot trial. All documents were stored on a user-restricted, secure password protected server in accordance with the University of Oxford data protection policies.

### Data extraction

One researcher extracted all data into a REDCap (REsearch Data Capture) database produced for this study. Double data extraction was carried out for 25 applications (25%) by a second researcher who was blinded to the date of funding call to which the application was submitted (pre- or post-2017).

Data was collected to describe characteristics of the application including the sample size, randomisation design and number of arms, whether progression criteria were reported and if so the characteristics of that progression criteria (including format, rationale or justification and whether it was reported who had decided on, or would assess, the criteria). We collected application outcomes at Stage 1 outline (rejected/proceed to Stage 2) and if applicable, Stage 2 (rejected/awarded funding).

We reviewed committee feedback to identify instances where progression criteria were mentioned, to determine whether committee feedback had led to inclusion of progression criteria or changes to the proposed progression criteria.

We documented whether the included applications that were awarded funding had yet been completed, and if so, whether they indicated that a future definitive trial was feasible. This information is readily collected as standard by RfPB through routine post close award monitoring.

### Data analysis

Descriptive statistics (proportions, median and IQR) were produced using Stata (v16.0; StataCorp) to describe the funding applications, their outcomes and the progression criteria where stipulated.

Although included applications had a funding decision date following publication of NIHR RfPB guidance to include progression criteria (July 2017), around half were submitted to funding calls launched before this guidance was published. We therefore conducted an additional post hoc analysis to compare whether applications with Stage 1 outline submitted to funding calls launched after July 2017 were more likely to include progression criteria compared to those submitted to funding calls launched before July 2017.

## Results

### Screening and inclusion of applications

**Fig. 1:**
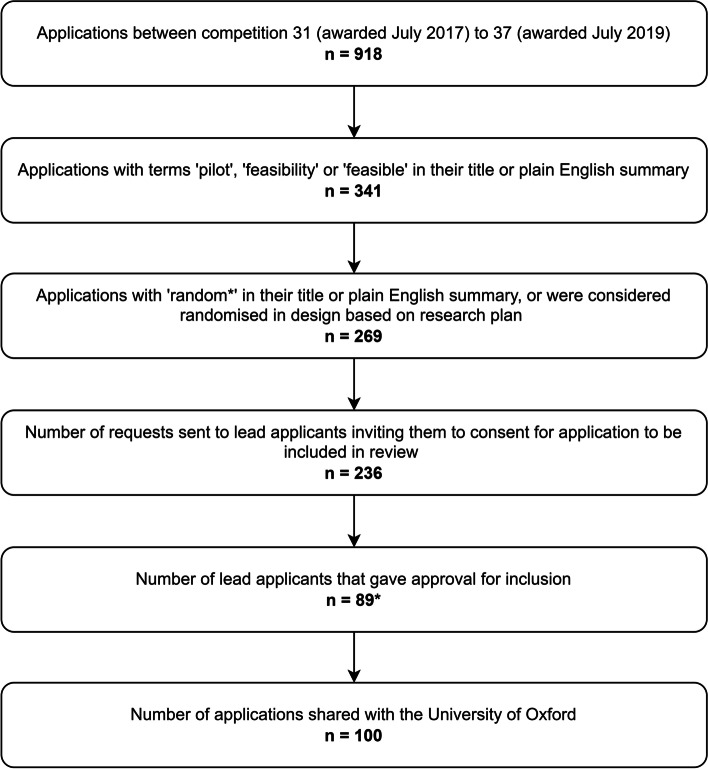
Flow chart to present screening and inclusion of applications. *Eleven applicants were lead applicants for more than one application

In total, 918 Stage 1 application outlines with funding decision made between July 2017 and July 2019 were identified from the database. Of these, 341 had the terms ‘pilot’, ‘feasibility’ or ‘feasible’ in their title or plain English summary. Two hundred sixty-nine included the term ‘random*’ in their title or plain English summary or were considered randomised in design based on initial review of their research plan. The 236 lead applicants of the 269 applications were contacted and invited to provide consent for their application to be included in the study. 89 lead applicants gave approval for 100 applications to be included, 14 applicants (lead applicants for 20 applications) responded but did not give approval, and 133 applicants (lead applicants for 149 applications) did not respond. In total, the research plans and committee feedback of 100 redacted applications were shared with the research team at the University of Oxford. This is summarised in Fig. 1.

### Characteristics of included applications

**Fig. 2:**
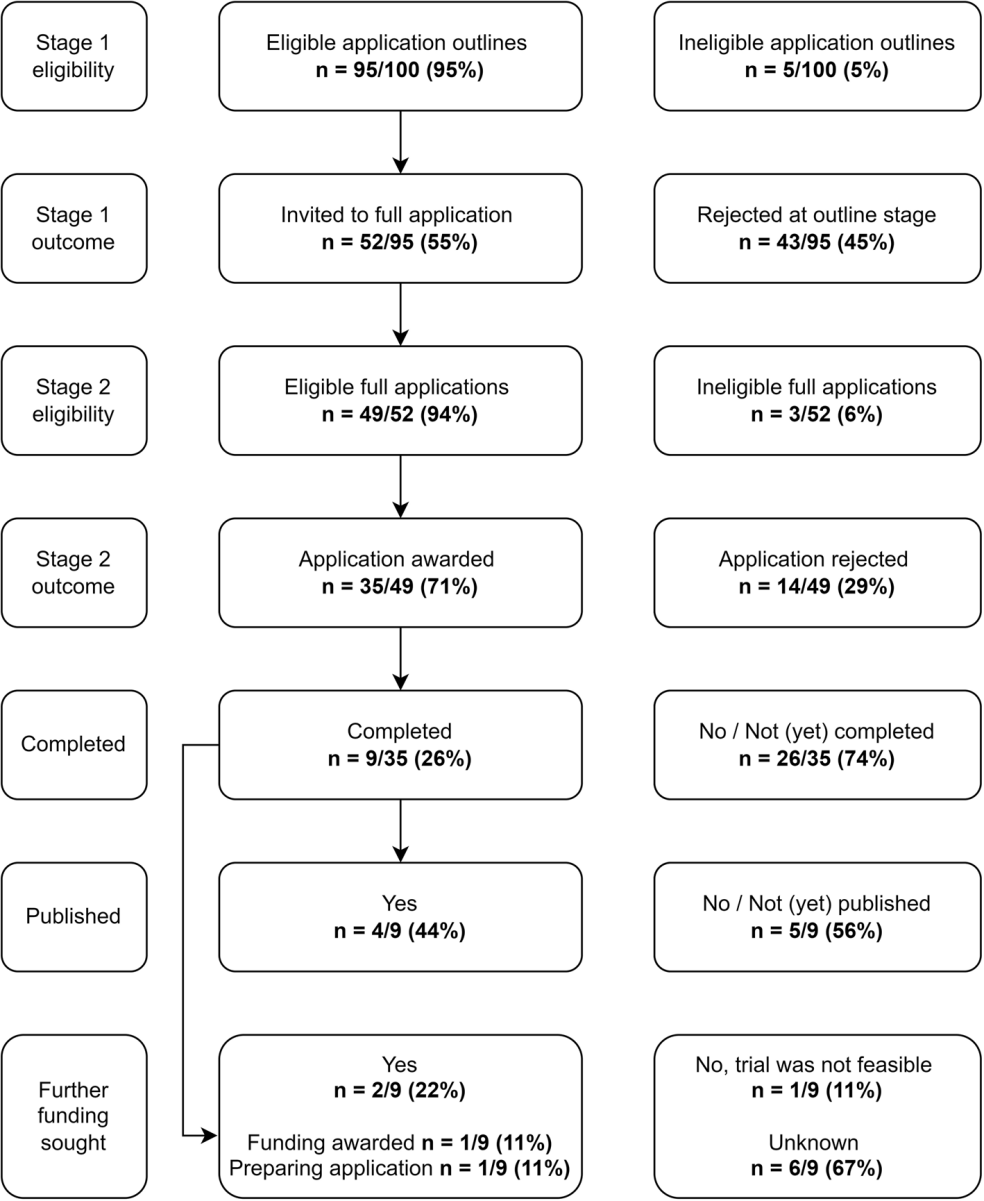
Flow chart to present funding outcomes of included applications

Of the 100 applications included in the original sample, five (5%) were determined to be ineligible at Stage 1 as they were single-arm studies and not randomised trials. Of the 95 eligible Stage 1 applications (outline stage), 52 (52/95, 55%) were invited to Stage 2 (full application) and 43 (43/95, 45%) were rejected at Stage 1. Three applications (3/52, 6%) were subsequently ineligible at Stage 2 because the randomised trial component had been dropped from the application (i.e. the full Stage 2 application was for a non-randomised pilot or single-arm feasibility study). Of the 49 eligible full Stage 2 applications, 35 (35/49, 71%) were awarded funding and 14 (14/49, 29%) were unsuccessful. Of the 35 that were awarded funding, at the time of data analysis nine had completed (9/35, 26%), four had been published (4/9, 44%), one had led to a further funding award for a definitive trial (1/9, 11%) and a funding application was being prepared for another (1/9, 11%), see Fig. 2.

Table 1 details the characteristics of included applications. Included applications assessed 23 therapeutic areas and six types of intervention. Of the 95 Stage 1 outline applications most were for multi-centre (69/95, 73%), two-arm (84/95, 88%) parallel randomised (84/95, 88%) trials. All but four Stage 1 application outlines provided a clear sample size, ranging from 20 to 250, with a median of *N*=60 (IQR 50–90). Most of the 49 eligible Stage 2 full applications were again for multi-centre (36/49, 73%), two-arm (44/49, 90%) parallel randomised (45/49, 92%) trials, assessing 21 different therapeutic areas and six types of intervention. The median sample size was again *N*=60 (range 25-800, IQR 50–80). At Stage 1, 80 applications (80/95, 84%) included plans to do qualitative research within or alongside the pilot trial. At Stage 2, 44 applications included a qualitative research component (44/49, 90%).

**Table 1:** Characteristics of included funding applications

	Outline application (Stage 1) (***n*** = 95)	Full application (Stage 2) (***n*** = 49)
**Therapeutic areas**^**a**^		
Oncology	12 (13%)	5 (10%)
Psychiatry/Ppychology	12 (13%)	7 (14%)
Paediatrics	10 (11%)	7 (14%)
Respiratory	7 (7%)	4 (8%)
Primary care	7 (7%)	1 (2%)
Gastroenterology/hepatology	6 (6%)	3 (6%)
Trauma	5 (5%)	4 (8%)
Other	36 (38%)	18 (37%)
**Intervention type**		
Drug	9 (9%)	4 (8%)
Surgery or procedure	15 (16%)	8 (16%)
Counselling, lifestyle or physiotherapy	55 (58%)	28 (57%)
Equipment	4 (4%)	3 (6%)
Medical device	1 (1%)	1 (2%)
Other	11 (12%)	5 (10%)
**Randomisation design**		
Parallel	84 (88%)	45 (92%)
Parallel + patient preference arms	2 (2%)	0 (0%)
Cluster	9 (9%)	4 (8%)
**Sample size**		
Sample size unclear in funding application	4	0
Min-max	20-250	25-800
Median	60	60
IQR	50-90	50-80
**Single/multi-centre**		
Single	19 (20%)	10 (20%)
Multi	69 (73%)	36 (73%)
Unclear	7 (7%)	3 (6%)
**Number of arms**		
2	84 (88%)	44 (90%)
>2	11 (12%)	5 (10%)
**Primary focus is the assessment of feasibility**		
Yes	94 (99%)	49 (100%)
No^b^	1 (1%)	0 (0%)
**Qualitative research conducted**		
Yes	80 (84%)	44 (90%)
No	15 (16%)	5 (10%)

Percentages might not add up to 100 due to rounding

95 Stage 1 applications were included, 52 were invited to Stage 2, three were ineligible and 49 were included

^a^Therapeutic areas that were given in ≥5 Stage 1 application outlines are listed; all others are categorised in ‘other’

^b^The primary focus of one study was only on safety rather than feasibility, this study was redesigned at Stage 2 as single arm proof of concept study (ineligible at Stage 2)

### Inclusion of progression criteria in studied applications

Table 2 describes the inclusion of progression criteria in funding applications, including a breakdown by funding stage and outcome. Just over half (48/95, 51%) of Stage 1 applications stipulated what the criteria for progression to a future definitive RCT would be. Of these, half (24/48, 50%) were invited to Stage 2. One application that stipulated progression criteria at Stage 1 was one of three applications that were ineligible at Stage 2 and so were removed from the subsequent analysis. At Stage 2 a larger proportion of applications stipulated progression criteria (73%, 36/49). Of these, 72% (26/36) were subsequently funded and 28% (10/36) were rejected. In total, 23 applications assessed at Stage 2 (23/49, 47%) included progression criteria at both Stage 1 and 2, and 13 applications that did not include progression criteria at Stage 1 did do so in the corresponding Stage 2 application (13/49, 27%). Of the 23 applications that stipulated progression criteria at both stages, over half (13/23, 57%) made changes to their progression criteria between Stages 1 and 2, for example altering specific numerical targets, or providing additional criteria.

**Table 2:** Inclusion and characteristics of progression criteria by application stage and outcome

	Outline application (Stage 1)			Full application (Stage 2)		
	Total	Invited	Rejected	Total	Awarded	Rejected
**Progression criteria reporting**	**(*n*** **= 95)**	**(*n*** **= 52)**^**a**^	**(*n*** **= 43)**	**(*n*** **= 49)**^**a**^	**(*n*** **= 35)**	**(*n*** **= 14)**
Yes	48 (51%)	24 (46%)^a^	24 (56%)	36 (73%)	26 (74%)	10 (71%)
Stipulated in Stage 1 and Stage 2	N/A	N/A	N/A	23 (65%)^a^	17 (65%)	6 (60%)
Stipulated in stage two only	N/A	N/A	N/A	13 (36%)	9 (35%)	4 (40%)
No	47 (49%)	28 (54%)	19 (44%)	13 (27%)	9 (26%)	4 (29%)
**Characteristics of progression criteria**	**(*n*** **= 48)**	**(*n*** **= 24)**	**(*n*** **= 24)**	**(*n*** **= 36)**	**(*n*** **= 26)**	**(*n*** **= 10)**
**Format**						
Distinct threshold / STOP-GO	18 (38%)	8 (33%)	10 (42%)	10 (27%)	9 (35%)	1 (10%)
Distinct threshold / STOP-GO +^b^	15 (31%)	8 (33%)	7 (29%)	11 (31%)	9 (35%)	2 (20%)
Traffic light system / STOP-AMEND-GO	5 (10%)	3 (13%)	2 (8%)	8 (22%)	3 (12%)	5 (50%)
Traffic light system / STOP-AMEND-GO +^b^	4 (8%)	2 (8%)	2 (8%)	5 (14%)	3 (12%)	2 (20%)
Non-numerical	6 (13%)	3 (13%)	3 (13%)	2 (6%)	2 (8%)	0 (0%)
**Presentation**						
Text	45 (94%)	23 (96%)	22 (92%)	32 (89%)	23 (88%)	9 (90%)
Table	3 (6%)	1 (4%)	2 (8%)	4 (11%)	3 (12%)	1 (10%)
**Areas of feasibility informing progression criteria**^**c**^						
Recruitment	41 (85%)	22 (92%)	19 (79%)	31 (86%)	21 (81%)	10 (100%)
Retention	27 (56%)	13 (54%)	14 (58%)	24 (67%)	16 (62%)	8 (80%)
Acceptability of intervention or trial (participants)	19 (40%)	9 (38%)	10 (42%)	15 (42%)	10 (38%)	5 (50%)
Data completion or missing data	16 (33%)	7 (29%)	9 (38%)	11 (31%)	7 (27%)	4 (40%)
Non/compliance or adherence (participants)	13 (27%)	4 (17%)	9 (38%)	13 (36%)	11 (42%)	2 (20%)
Consent or refusal rate	9 (19%)	4 (17%)	5 (21%)	6 (17%)	6 (23%)	0 (0%)
Acceptability of intervention or trial (non-participants)	8 (17%)	5 (21%)	3 (13%)	11 (31%)	8 (31%)	3 (30%)
Intervention fidelity	7 (15%)	1 (4%)	6 (25%)	9 (25%)	7 (27%)	2 (20%)
Safety or adverse events	6 (13%)	5 (21%)	1 (4%)	7 (19%)	6 (23%)	1 (10%)
Determine/estimate definitive trial sample size	6 (13%)	4 (17%)	2 (8%)	4 (11%)	4 (15%)	0 (0%)
Completion or withdrawal	5 (10%)	2 (8%)	3 (13%)	4 (11%)	4 (15%)	0 (0%)
Randomisation	5 (10%)	1 (4%)	4 (17%)	3 (8%)	2 (8%)	1 (10%)
Other	35 (73%)	18 (75%)	17 (71%)	31 (86%)	23 (88%)	8 (80%)
**Qualitative research informs progression criteria**						
Yes	20 (42%)	10 (42%)	10 (42%)	18 (50%)	12 (46%)	6 (60%)
No	18 (38%)	8 (33%)	10 (42%)	14 (39%)	11 (42%)	3 (30%)
No qualitative research component	10 (21%)	6 (25%)	4 (17%)	4 (11%)	3 (12%)	1 (1%)
**Justification or rationale for progression criteria given**						
Yes	9 (19%)	3 (13%)	6 (25%)	7 (19%)	5 (19%)	2 (20%)
For some criteria	7 (15%)	5 (21%)	2 (9%)	5 (14%)	4 (15%)	1 (10%)
No	32 (67%)	16 (67%)	16 (67%)	24 (67%)	17 (65%)	7 (70%)
**Application details who decided on progression criteria**						
Yes	1 (2%)	0 (0%)	1 (4%)	1 (3%)	0 (0%)	1 (10%)
No	47 (98%)	24 (100%)	23 (96%)	35 (97%)	26 (100%)	9 (90%)
**Application details who will assess progression criteria**						
Yes	8 (17%)	3 (13%)	5 (21%)	9 (25%)	6 (23%)	3 (30%)
No	40 (83%)	21 (88%)	19 (79%)	27 (75%)	20 (77%)	7 (70%)

Percentages might not add up to 100 due to rounding

95 Stage 1 applications were included, 52 were invited to Stage 2, three were ineligible and 49 were included

^a^Three applications that were invited following Stage 1 were subsequently ineligible at Stage 2; One of these applications included progression criteria at Stage 1

^b^+ indicates additional considerations specified that are not in a STOP-GO or STOP-AMEND-GO format

^c^Areas of feasibility that were given in ≥5 Stage 1 application outlines are listed; all others are categorised in ‘other’; multiple areas of feasibility might inform progression criteria in individual applications

There was little difference between progression criteria reporting and funding outcomes at each application stage. At Stage 1, 46% (24/52) of invited applications included progression criteria, which was less than 56% (24/43) of rejected applications that included progression criteria. These proportions were more equal at Stage 2, where 74% (26/35) of awarded applications included progression criteria compared to 71% (10/14) rejected applications.

### Characteristics of progression criteria where stipulated

Table 2 also describes the characteristics of included progression criteria. Most applications provided progression criteria in a stop-go format where distinct thresholds were given against which feasibility would be assessed (Stage 1 33/48, 69%; Stage 2 21/36, 58%). In both instances, around half also reported additional considerations, often non-numerical, that would inform the interpretation of pilot trial findings (denoted in the table as ‘distinct threshold/STOP-GO+’, Stage 1 15/33, 45%; Stage 2 11/21, 52%). A larger proportion of applications at Stage 2 reported progression criteria in a ‘traffic light system/STOP-AMEND-GO’ format, with or without additional considerations, compared to applications at Stage 1 (Stage 1 9/48, 19%; Stage 2 13/36, 36%). At both Stage 1 and 2, some applications opted to stipulate progression criteria in a non-numerical format (Stage 1 6/48, 13%; Stage 2 2/36, 6%), for example ‘progression to a future RCT will be discussed with the Trial Steering Committee (TSC) at the end of the study and will be informed by the observed recruitment rate, and the number of participants retained at 12-months.’

The most frequent uncertainties about feasibility that informed progression criteria were recruitment (Stage 1 41/48, 85%; Stage 2 31/36, 86%), retention (Stage 1 27/48, 56%; Stage 2 24/36, 67%) and acceptability of the trial or intervention to participants (Stage 1 19/48, 40%; Stage 2 15/36, 42%). Participant noncompliance (adherence) (Stage 1 13/48, 27%; Stage 2 13/36, 36%), and data completion (Stage 1 16/48, 33%; Stage 2 11/36, 31%) also often contributed to progression criteria.

Progression criteria in 20 applications at Stage 1 were informed by the findings of qualitative research (20/48, 42%) with half invited to Stage 2 (10/20, 50%) and half rejected (10/20, 50%). Half of the applications assessed at Stage 2 included progression criteria that would be informed by qualitative research (18/36, 50%), 12 were awarded funding (12/18, 66%) and six were rejected (6/18, 33%).

One-third of applications reported justification or rationale for choice of all or some of the specified progression criteria (Stage 1 16/48, 33%; Stage 2 12/36, 33%). At Stage 1 half of the applications that provided some rationale or justification for criteria were invited to Stage 2 (8/16, 50%). Of the 12 applications that provided some rationale or justification at Stage 2, nine (9/12, 75%) were funded and three (3/12, 25%) were rejected.

One application at Stages 1 and 2 detailed who had decided on progression criteria (Stage 1 1/48, 2%; Stage 2 1/36, 3%). Eight applications at Stage 1, and nine at Stage 2 detailed who would be involved in assessing progression criteria (Stage 1 8/48, 17%; Stage 2 9/36, 27%). In all instances, a Trial Steering Committee would be involved in assessing progression criteria (Stage 1 8/8, 100%; Stage 2 9/9, 100%). Other parties included the Trial Management Group (Stage 1 2/8, 20%; Stage 2 3/9, 33%), Participant Representatives (Stage 1 1/8 13%), and a Data Monitoring Committee (Stage 2 1/9, 11%).

### RfPB committee feedback for included applications

Table 3 describes funding committee feedback in relation to progression criteria. At both Stage 1 and Stage 2, over 20% of application committee feedback explicitly mentioned progression criteria (Stage 1 22/95, 23%; Stage 2 11/49, 22%). At Stage 1, most often feedback implied that progression criteria were not stipulated in the funding application (Stage 1 11/22, 50%; Stage 2 4/11, 36%). Committee feedback for an additional eight Stage 1 and three Stage 2 applications requested further detail or clarity for progression criteria, e.g. the addition of numerical thresholds where these were not included or for progression criteria to be expanded (Stage 1 8/22, 36%; Stage 2 3/11, 27%). There were also instances where committee feedback queried rationale or justification for stated progression criteria, e.g. why a certain target had been set (Stage 1 3/22, 14%; Stage 2 4/11, 36%). At both Stage 1 and 2, there were instances where applications did not stipulate progression criteria and no reference to progression criteria was included in the committee feedback (Stage 1 35/95, 37%; Stage 2 9/49, 18%).

**Table 3:** Committee feedback in relation to progression criteria by application stage and outcome

	Outline application (Stage 1)			Full application (Stage 2)		
	Total	Invited	Rejected	Total	Awarded	Rejected
**Funding committee feedback explicitly mentions progression criteria**	**(*n*** **= 95)**	**(*n*** **= 52)**	**(*n*** **= 43)**	**(*n*** **= 49)**	**(*n*** **= 35)**	**(*n*** **= 14)**
Yes	22 (23%)	15 (29%)	7 (16%)	11 (22%)	9 (26%)	2 (14%)
Progression criteria stipulated	10 (45%)	7 (47%)	3 (43%)	7 (64%)	5 (56%)	2 (100%)
Progression criteria not stipulated	12 (55%)	8 (53%)	4 (57%)	4 (36%)	4 (44%)	0 (0%)
No	73 (77%)	37 (71%)	36 (84%)	38 (78%)	26 (74%)	12 (86%)
Progression criteria stipulated	38 (52%)	17 (46%)	21 (58%)	29 (76%)	21 (81%)	8 (67%)
Progression criteria not stipulated	35 (48%)	20 (54%)	15 (42%)	9 (24%)	5 (19%)	4 (33%)
**Details of feedback where stipulated**	**(*n*** **= 22)**	**(*n*** **= 15)**	**(*n*** **= 7)**	**(*n*** **= 11)**	**(*n*** **= 9)**	**(*n*** **= 2)**
Feedback implies progression criteria were not stipulated	11 (50%)	7 (47%)	4 (57%)	4 (36%)	4 (44%)	0 (0%)
Feedback requests further detail/clarity e.g. numerical thresholds	8 (36%)	5 (33%)	3 (43%)	3 (27%)	3 (33%)	0 (0%)
Feedback relates to rationale or justification for progression criteria	3 (14%)	3 (20%)	0 (0%)	4 (36%)	2 (22%)	2 (100%)

95 Stage 1 applications were included, 52 were invited to Stage 2, three were ineligible and 49 were included

### Examining the effect of funder guidance

Of the 100 included applications, 49 were submitted to funding calls launched prior to July 2017 (the date guidance was first published to include progression criteria in NIHR RfPB applications). Table 4 presents a pre-post comparison of progression criteria inclusion in relation to this date.

**Table 4:** Inclusion of progression criteria by funding call submission pre- and post-July 2017

Progression criteria stipulated	Outline application (Stage 1)		Full application (Stage 2)	
	Pre-July 2017 (***n*** = 47)	Post-July 2017 (***n*** = 48)	Pre-July 2017 (***n*** = 27)	Post-July 2017 (***n*** = 22)
Yes	17 (36%)	31 (65%)	14 (52%)	22 (100%)
No	30 (64%)	17 (35%)	13 (48%)	0 (0%)

95 Stage 1 applications were included, 52 were invited to Stage 2, three were ineligible so 49 were included

Of the 95 applications eligible at Stage 1, 47 (49%) were submitted to funding calls launched pre-July 2017, and 48 (51%) were submitted to funding calls launched post-July 2017. The proportion of Stage 1 applications that included progression criteria increased following July 2017 (from 17/47, 36% to 31/48, 65%). Of the 49 applications eligible at Stage 2, 27 (55%) were submitted to funding calls launched pre-July 2017, and 22 (45%) were submitted to funding calls launched after July 2017. All Stage 2 applications submitted to funding calls launched after July 2017 included progression criteria in their research plans (22/22, 100%), compared to just over half of those submitted before July 2017 (14/27, 52%).

## Discussion

### Statement of principal findings

This study presents the findings from an investigation of the research plans and committee feedback of 100 funding applications for randomised pilot trials submitted to the UK NIHR RfPB funding stream between July 2017 and July 2019. In total, 95 of the 100 Stage 1 application outlines assessed were eligible at Stage 1, 52 were invited to full Stage 2 application, of which 49 were eligible at Stage 2. Just over half of the application research plans assessed at Stage 1 outline and just under three quarters of those assessed at full Stage 2 application included progression criteria. Our findings indicate that the publication of funder guidance in July 2017 for progression criteria to be included in applications for pilot and feasibility trials, and prompts from funding committee members, promoted the inclusion of progression criteria across both application stages.

### Strengths and weaknesses of this study

This is the first study of its type to provide data on progression criteria for external pilot RCTs submitted to a large research funder in the UK. This research serves as an example of how researcher-funder collaboration can enhance trial methodology research. This collaboration allowed us to include both successful and unsuccessful funding applications as a research data source, and RfPB Programme Managers had a crucial role in obtaining consent from researchers for the inclusion of their application. However, we acknowledge that obtaining consent may have introduced bias, with researchers who were awarded funding perhaps more likely to give consent than those who were not. This might explain why the success rate of included applications is higher than the typical success rate of RfPB funding applications assessed at Stage 1 (around 20%) [10].

The research data was also limited to the redacted research plan section of the funding application, and although unlikely, other parts of the application might have contained data that was relevant to address the research aims. In addition, we acknowledge that our assessment of funding committee feedback was subjective. It was sometimes difficult to determine whether funding committee members were or were not requesting the addition of progression criteria, often because the language used was ambiguous. For example, reference to progression criteria lacking ‘detail’ was made both when applications did and did not include progression criteria. How funding committee members can ensure feedback clarity is an interesting research question to be addressed.

Although inclusion of a recent sample of funding applications is a strength of this study, this meant that we were unable to collect post funding award outcomes for a large proportion of those that were funded. Many might simply not have had enough time to proceed to completion or could have faced delays due to Covid-19. This limits any conclusions we can draw about whether applications with clearly defined progression criteria are more likely to lead to a future definitive RCT. A longer-term follow-up of these applications to determine how many progressed to further research funding awards would add value to these findings.

### Findings in context and implications for clinicians and policymakers

We found that a higher proportion of full Stage 2 applications stipulated progression criteria compared to Stage 1 outlines which might be expected given the research plan word limit at Stage 1 [11]. We also found that the proportions of applications reporting progression criteria were similar between those that were invited or awarded, and those rejected at each stage. This indicates that although the reporting of progression criteria improved between Stages 1 and 2, inclusion of progression criteria did not necessarily mean that applications were more likely to be invited or awarded funding. Instead, this improvement in reporting was more likely due to funding committee members requesting the inclusion of progression criteria where they were not stipulated. This finding might be surprising considering the 2016 CONSORT guidance outlined that a decision process about how to proceed to a future definitive RCT, which might involve formal progression criteria, should be built into pilot trial design [12]. Current NIHR RfPB guidance states that ‘a clear route (e.g. progression criteria) should be included in the research plan’ of feasibility study funding applications [2]. The guidance also advises that ‘RfPB committees consider the pathway to RCT as part of their assessment’, yet over one-third of Stage 1 application outlines submitted following the publication of this guidance did not include progression criteria.

Where stipulated, progression criteria most often followed a stop-go format, with many applications also stipulating additional factors that researchers would consider when determining feasibility. The stop-amend-go format, which is recommended for progression criteria in RCTs with internal pilot phases [7], was less often used. However, the proportion of applications that opted for a stop-amend-go format increased between Stages 1 and 2. We also identified several funding applications that included non-numerical progression criteria, however, this reduced between Stages 1 and 2, either because the application was rejected or following a request of the funding committee for further detail or clarity around progression criteria, such as the addition of specific quantifiable thresholds.

We identified recruitment as the most common indicator of feasibility to inform progression criteria, followed by retention. This supports the suggestion that researchers conducting feasibility studies might focus more on uncertainties that are perceived to be important to research funders, such as recruitment, rather than others that are equally important to trial success, such as intervention fidelity [13]. Although we did identify instances where funding committee members queried rationale for choice of progression criteria, our findings suggest that how progression criteria have been developed (e.g. what rationale they are based on and who decided on them) might be less important to researchers and funding committee members when assessing funding applications. This information, which is also not often reported in pilot trial publications [14], was rarely included in this sample of funding applications.

## Conclusions

Although inclusion of progression criteria in pilot trial funding applications submitted to NIHR RfPB has increased following the publication of guidance to do so, some applicants still did not include progression criteria in their Stage 1 application outline. We propose that this could be due to a lack of clarity for what constitutes clear progression criteria for external pilot trials. Consideration should be given to develop best practice recommendations for progression criteria in external pilot trials to support researchers submitting future funding applications, and funding committee assessment.

## Data Availability

A Data Sharing Agreement was produced prior to this study which stipulates that the included applications cannot be shared. This is to maintain the anonymity of the researchers who gave approval for their funding application to be included in this study.

## Abbreviations

CONSORT: Consolidated Standards of Reporting Trials
HTA: Health Technology Assessment
NIHR: National Institute for Health Research
RCT: Randomised controlled trial
REDCap: REsearch Data Capture
RfPB: Research for Patient Benefit
TSC: Trial Steering Committee
UK: United Kingdom
